# Micro-computed tomographic analysis of the morphology of maxillary canines

**DOI:** 10.1038/s41598-024-84877-0

**Published:** 2025-02-03

**Authors:** Thomas Gerhard Wolf, Theodora Rempapi, Niccolò Giuseppe Armogida, Gianrico Spagnuolo, Andrea Lisa Waber

**Affiliations:** 1 Department of Periodontology and Operative Dentistry, University Medical Center of the Johannes Gutenberg, University Mainz, Augustusplatz 2, Mainz, 55131 Germany; 2 Department of Restorative, Preventive and Pediatric Dentistry, School of Dental Medicine, University of Bern, Freiburgstrasse 7, 3010 Bern, Switzerland; 3 Department of Neurosciences, Reproductive and Odontostomatological Sciences, University of Naples “Federico II”, Napoli, Italy

**Keywords:** Internal morphology, Maxillary canine, Micro-computed tomography, Root canal configuration, Swiss-German, Anatomy, Dental pulp, Dentine, Enamel, Dental public health, Dentistry, Endodontics, Root canal treatment

## Abstract

Objective: This study aimed to examine the morphology of maxillary canines (MxCs) by means of micro-computed tomography (micro-CT). Materials and methods: The root canal configurations (RCCs) of 97 maxillary canines of a mixed Swiss-German population were analyzed using micro-CT. After representing the internal morphology by 3-D software imaging, the RCC results were described using a four-digit system code indicating the main root canal from coronal to apical thirds and the main foramina number. Results: The most frequently observed RCCs of the MxC of the Swiss-German population were 1-1-1/1 (77.3%), followed by 1-1-1/2 (14.4%), 1-1-2/2 (4.1%), and finally 1-1-1/3 and 1-2-1/1 with 2.1% each. One physiological foramen was observed in 79.4% of the samples, two in 18.6%, and only 2.1% had three foramina. In 52.6% of the MxC samples, accessory and connecting canals were identified, with the majority located in the apical third of the root. Conclusions: This study contributes detailed information about the RCCs of MxC. The most prevalent RCC is 1-1-1/1, with accessory or connecting canals present in over half of the samples. However, it is noteworthy that in more than one-fifth of the examined samples, a particularly challenging RCC was observed. This should be considered when selecting treatment techniques. Clinical relevance: This study presents the root canal configurations in maxillary canines of a Swiss-German population and emphasizes the importance of influencing endodontic treatment decisions and outcomes.

## Introduction

A detailed knowledge of the three-dimensional internal root canal morphology is a requirement for successful nonsurgical or surgical endodontic treatment^1^. The expanded awareness of the complexity of the root canal anatomy simplifies the planning and implementation of endodontic therapy and reduces the possibility of iatrogenic errors^2,3^. The existence of various morphological structures of the root canal system makes it necessary to diagnose and evaluate each case individually. According to different authors, the most frequent root canal configuration (RCC) of maxillary canine is a single root canal from the pulp chamber to the apex (1-1-1/1)^1^. Various ex vivo research methods have been used to understand the morphology of root canal systems, such as the staining and clearing technique^1^, or magnification^4^. CBCT examinations are well established for in vivo studies^5–7^, while micro-computed tomography (micro-CT) analysis is considered the gold standard for ex vivo studies^8,9^. Micro-CT imaging, combined with rendering and 3D software analysis, offers a non-invasive and reproducible technique for preserving tooth structure and gaining insight into the complex internal features of the root canal system^8,10^. The classifications of root canal configurations proposed by Weine et al.^11^ and Vertucci^1^ describe possible variations of the root canal system; unfortunately, they cannot respond to the morphological intricacies of some root canals. However, to the best of the authors’ knowledge, there is no precise analysis of the expected internal root canal morphology or RCCs of maxillary canines in a Swiss-German population in the literature to date^12^. The hypothesis of the current study was that various RCCs occur in a Swiss-German population. Therefore, the aim of this study was to investigate the internal morphology of maxillary canines in this population using micro-CT, classify the observed RCC variations with a 4-digit code system, and provide findings to assist clinicians in making more informed treatment decisions that lead to better outcomes.

## Materials and methods

### Tooth selection

A total of 97 extracted human permanent maxillary canines (MxCs) were collected from dental clinics and dentists in Switzerland and Germany for reasons unrelated to the present investigation. The samples were cleaned with manual scalers, curettes (HuFriedyGroup EverEdge™ 2.0; Chicago, Illinois, USA), and ultrasonic instruments (Pieton 150; EMS Dental, Nyon, Switzerland) to completely remove any remaining calculus and soft tissue. After careful cleaning, the teeth were placed in an ultrasonic bath with 3% hydrogen peroxide for one hour and then stored in a 2% chloramine solution until the investigation was performed. All teeth examined in this study were declared “excess material” and could therefore be used for scientific purposes without the need for additional ethics committee approval (Contract General Terms [AVB], § 14 Organ explantation/further use of body material, Status: 1 April 2017). All patients providing samples gave their written consent to use their biomaterial for research purposes. The teeth were selected by two independent examiners and had to be clearly identified as MxCs. The sample size was calculated based on teeth extracted for reasons unrelated to this study and meeting the inclusion criteria described below, over a period of six months, from June 1 to December 31, 2022. A proportional test with a 95% confidence level and an expected prevalence of 90% was conducted. An additional 5% of teeth were included to account for potential artifacts that could render analysis impossible or lead to failure, resulting in a final sample size of 97 teeth^13^. The MxCs had to meet the following inclusion criteria: complete coronal and root development, no signs of root fracture or resorption, no radicular or coronal caries, and no endodontic treatment (12.5x, ZEISS S100/OPMI pico; Carl Zeiss Meditec AG, Jena, Germany). If the criteria were not met, the teeth were excluded and could not be considered in the study.

### Micro-computed tomography investigation

The MxCs were scanned using a previously established method^14,15^ at an isotropic resolution of 16 μm in a desktop micro-CT unit (µCT 40; SCANCO Medical AG, Brüttisellen, Switzerland) at settings of 70 kV and 114 mA, resulting in 800–1200 slices per tooth. To identify different tooth structures, the images obtained were visualized and displayed with dummy colors and 3D reconstructions of the micro-CT scans with the use of a specialized imaging software (VGStudio Max 2.2; Volume Graphics, Heidelberg, Germany). In particular, the pulp chamber and the root canal system were colored red, the coronal enamel white, and the root and dentin areas transparent gray. Hydroxyapatite phantoms serve as reference samples with variable thickness, density and spatial orientation. These are measured once a week for calibration and standardization and the results are logged.

### Root canal configuration method

The root canal system is described by the classification system according to Briseño Marroquín et al.^16^ with the use of a four-digit system code. The first, second and third digits represent the coronal, middle and apical thirds, respectively. Each digit represents the number of root canals at the coronal limit of the corresponding third. The fourth digit is separated by a slash (/) and indicates the number of physiological foramina at the apex. Physiological foramina were defined as those belonging to the same root canal and having a diameter of at least 0.1 mm^16^. Foramina with a diameter less than 0.1 mm were defined as accessory foramina. In addition, the number of accessory foramina and connecting canals observed using micro-CT were also recorded and classified according to their localization in the root thirds (apical, middle, or coronal). A connecting canal connects the same or another canal without passing through the surrounding periapical tissue. An accessory canal is either blind-ending or opens into an accessory foramen. The descriptive results of the current study are presented as absolute and relative values.

## Results

**Fig. 1:**
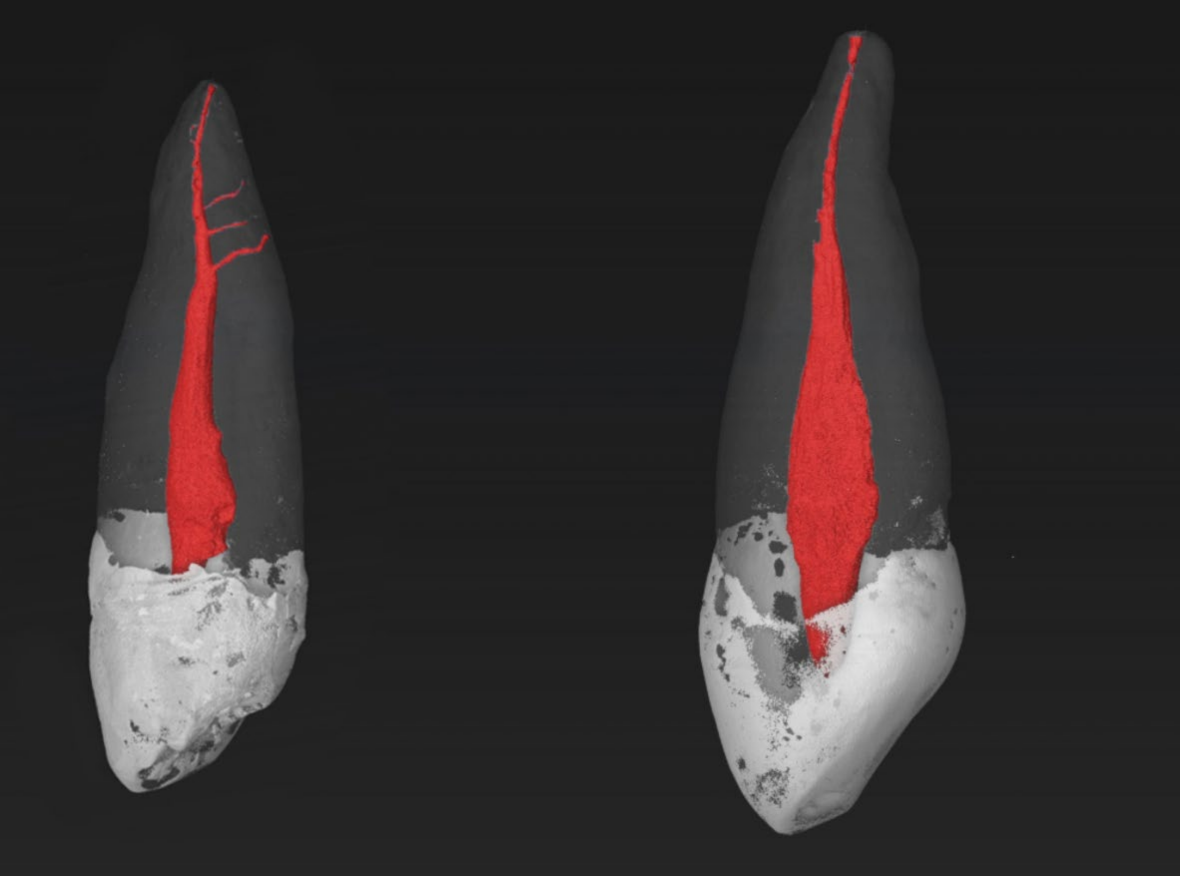
Micro-CT images of MxCs with a RCC of 1-1-1/1. Accessory canals can be observed in the apical third of the left tooth.

**Fig. 2:**
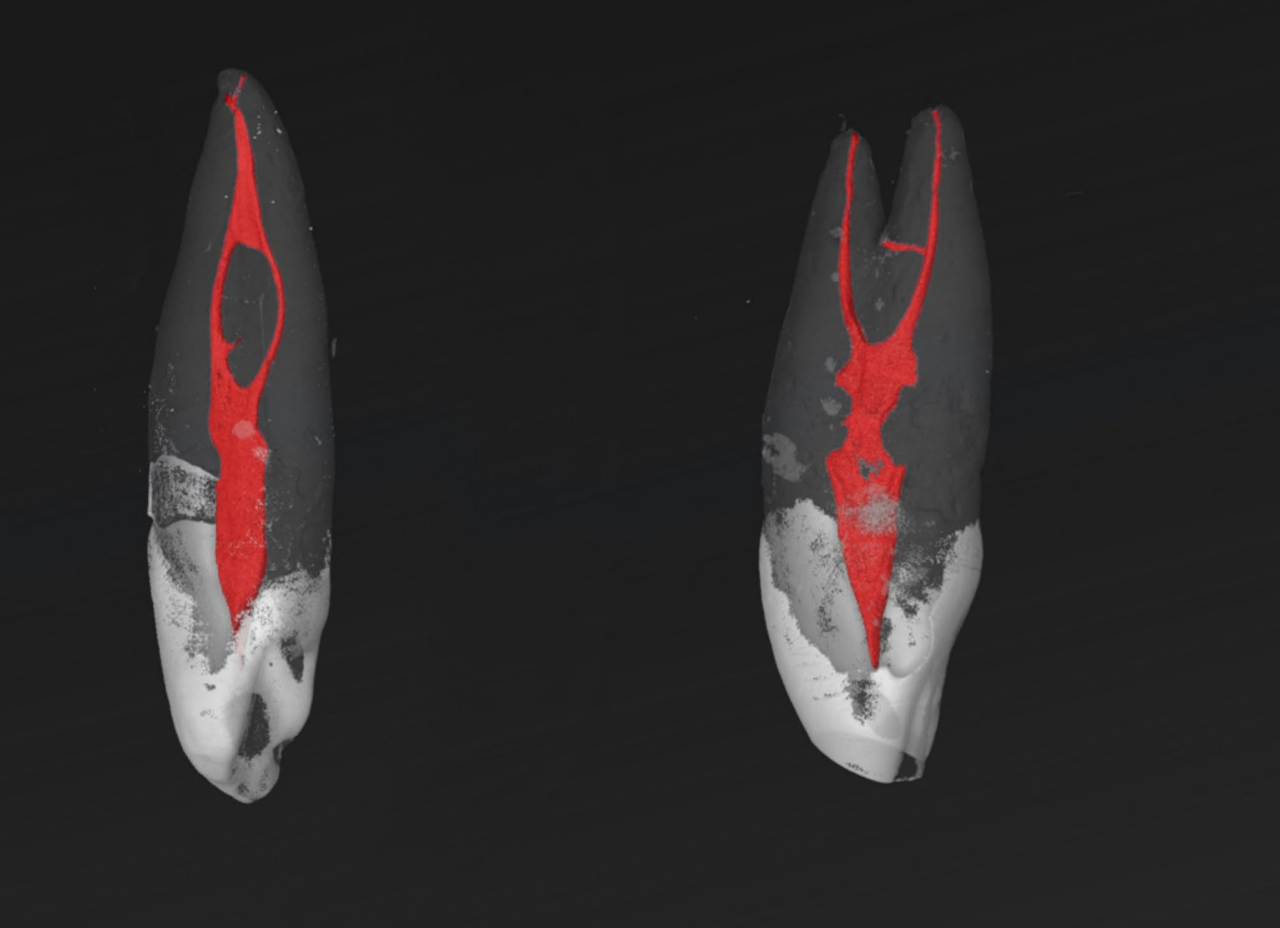
Micro-CT images of MxCs with RCCs of 1-2-1/1 (left) and 1-1-2/2 (right), whereas the right tooth had two roots. An accessory canal can be observed in the apical third of both teeth.

**Fig. 3:**
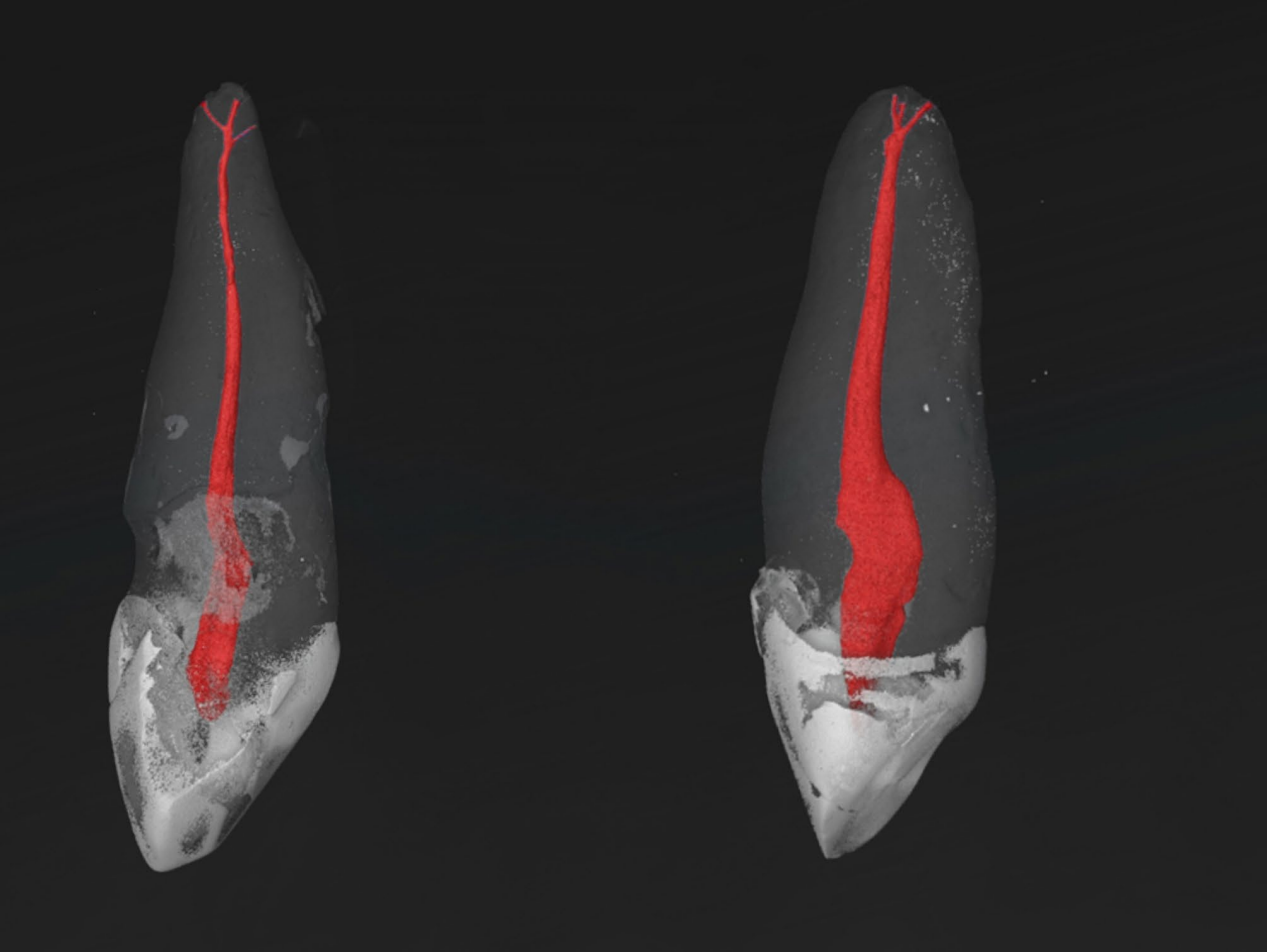
Micro-CT images of two MxCs with a RCC of 1-1-1/3. Dental structures are color-coded: the pulp and root canal system with red, enamel with white and dentin with transparent gray.

**Fig. 4:**
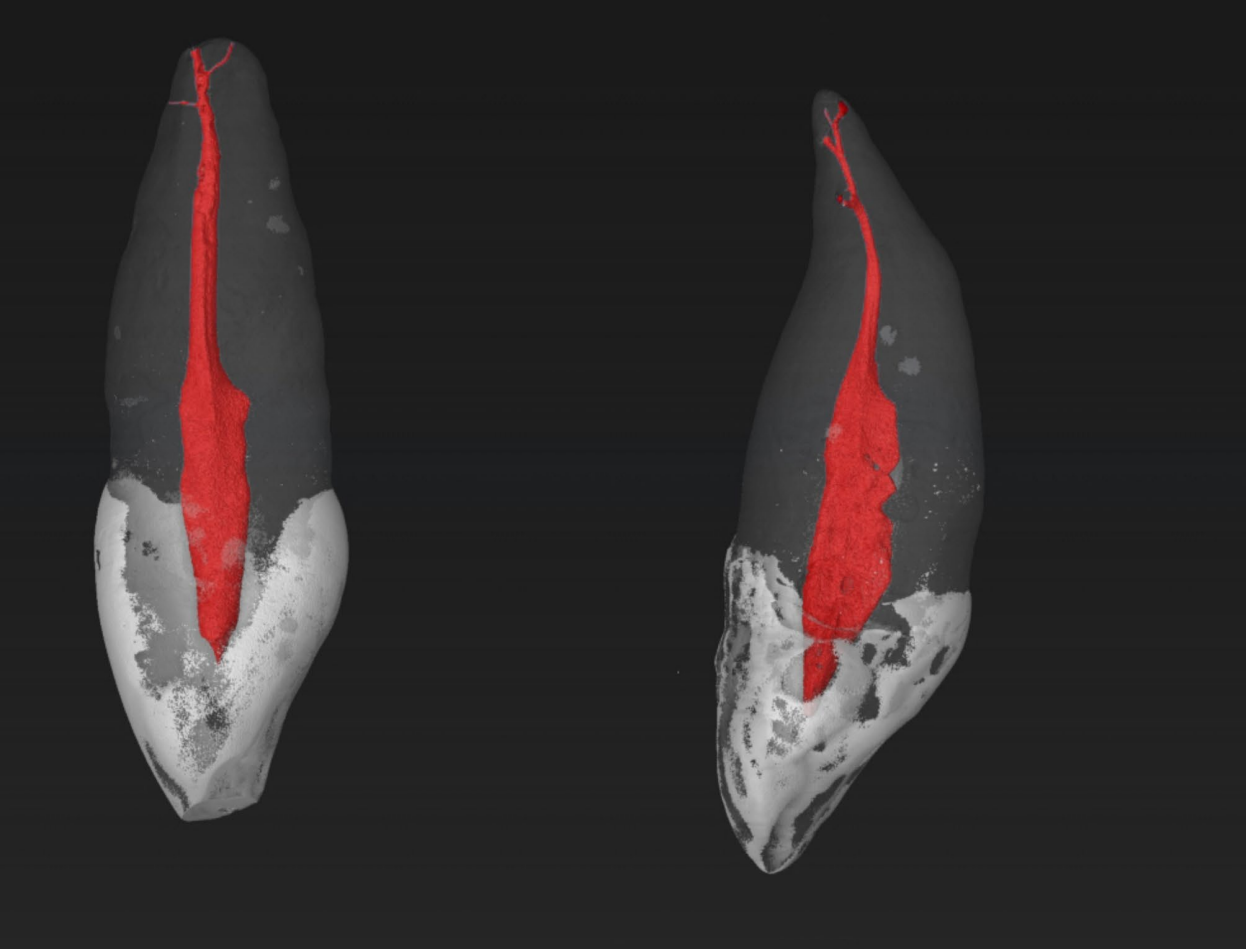
Micro-CT images of MxCs with RCCs of 1-1-1/1 (left) and 1-1-1/2 (right) RCC. Two main foramina and two accessory canals can be observed in the apical thirds of both teeth.

The root canal configuration results of 97 maxillary canines are shown in Table 1. All examined samples were single-rooted. The most frequently observed root canal configurations were 1-1-1/1 (77.3%), followed by 1-1-1/2 (14.4%), and 1-1-2/2 (4.1%), two other RCCs (1-1-1/3 and 1-2-1/1) were detected with a percentage of 2.1% (Figs. 1, 2, 3 and 4). The results of the main physiological foramina frequency are shown in Table 2. One physiological foramen was examined 79.4% of the in the MxCs. Two main foramina were observed in 18.6% and only two samples (2.1%) had three foramina. Accessory and connecting canals were observed in the apical third (50.5%) and in the middle third (2.1%) of the root, while 47.4% of the MxCs had no accessory canals (Table 3).

**Table 1:** Root canal configurations of maxillary canines by means of micro-CT.

Root canal configuration			*n*	%
Briseño Marroquín et al.^15^	Vertucci^1^	Weine et al.^12^		
1-1-1/1	I	I	75	77.3
1-1-1/2		II	14	14.4
1-1-1/3			2	2.1
1-2-1/1	III		2	2.1
1-1-2/2	V		4	4.1
**Total**			**97**	**100.0**

**Table 2:** Absolute (n) and mean (%) frequency of main physiological foramina.

Main physiological foramina		
	*n*	%
/1	77	79.4
/2	18	18.5
/3	2	2.1
**Total**	**97**	**100.0**

**Table 3:** Number (n) and mean (%) frequency of accessory and connecting canals observed in the coronal, middle and apical thirds of maxillary canines using micro-CT.

Accessory and connecting canals		
	*n*	%
None	46	47.4
Coronal	0	0.0
Middle	2	2.1
Apical	49	50.5
**Total**	**97**	**100.0**

## Discussion

The objective of this study was to examine the root canal configuration of maxillary canines (MxCs) with a solid sample size to allow for reliable statistical analysis by using micro-computed tomography (micro-CT). The internal morphology and the root canal configuration have been the subject of many different examination methods over the years. Earlier studies used methods such as conventional two-dimensional radiographs^17^, clearing and staining with ink or dye^4^, and cross-sectional or magnification^18^ to examine internal tooth structures. Radiographs are difficult to reproduce and have a relatively low resolution, which means that very fine structures such as accessory canals cannot be accurately depicted. Decalcification and cross-sectioning are very time-consuming, the samples are cut and destroyed, and accurate reconstruction of the internal structures is difficult due to the thickness of the slices. Therefore, it is not surprising that with the advancement of three-dimensional imaging techniques, these methods are being replaced by minute and more objective methods for studying root canal morphology^8,10,14^.

Cone beam computed tomography (CBCT) has been used extensively in recent studies of the root canal morphology of maxillary canines with relatively large sample sizes^6,7,19^. When comparing the internal tooth morphology information from an *in vitro* micro-CT study with in vivo research methods such as CBCT, micro-CT is considered to provide more details and fine ramifications. The data obtained by micro-CT imaging simplifies the evaluation process and provides superior quantitative and qualitative information about the samples compared to alternative techniques^8,16^. However, due to the high radiation exposure, micro-CT is currently not suitable for in vivo clinical use in humans. Micro-CT analysis is a widely accepted method with the advantage of being non-invasive, non-destructive, and reproducible, and can now be considered the gold standard for ex vivo examination of root canal morphology^5,8^. To the best of the authors’ knowledge, only one other study has examined the root canals of MxCs by using micro-CT^19^.

The RCC classification systems proposed by Vertucci^1^, which distinguishes eight different types, and Weine et al.^11^, which classifies three different types of RCCs, have been widely reported in the literature. However, various computerized imaging techniques such as micro-CT, have improved the ability to image the finest details of root canals that are difficult to accurately classify with the previously mentioned RCC systems. Micro-CT imaging allows better visualization of the smallest tooth structures, therefore Briseño Marroquin et al.’s classification system^16^ is more appropriate for describing RCCs in the present report. The number of roots is defined in the apical, middle, and cervical thirds, and the number of physiological foramina is described by a four-digit code system^16^.

To the best of the authors knowledge, no evaluation of the root canal morphology of the MxC by micro-CT in a Swiss-German population has been performed yet. However, Plascencia et al.^19^ evaluated 32 MxC by micro-CT in a Western Mexican population. In this study, the 1-1-1/1 RCC was the most common at 93.7%, which is consistent with the present study where 1-1-1/1 RCC was also the most frequently observed at 77.3%^4^ and previous investigation using other methods^1,6,9,17,20–24^. The second most frequent RCC in the present study was the 1-1-1/2 (14.4%), which describes one root canal ending with two physiological foramina. To the best of the authors’ knowledge was this configuration not previously described in the literature of studies investigating MxCs. Furthermore, the RCCs 1-1-2/2 (4.1%), 1-1-1/3, and 1-2-1/1 (2.1% each) were identified in the present study. These results are consistent with previous reports that have described RCC frequencies of 1-1-2/2 ranging from 0.1–2.4%^7,8,19,20,23^ , and 1-2-1/1 in 0.1–11.6%^4^. A recent systematic review with meta-analysis has also shown that the most common RCC was 1-1-1/1 with approximately 75.4–100%, complex RCCs such as 2-2-1/1 and 1-2-1/1 were more common in men, while 2-2-2/2 was more often observed in women^12^. However, it must be considered that these sex-specific differences relate to studies from Asia and Europe, while other geographic regions have not yet been sufficiently studied in terms of age and sex.

The differences in results between studies can be attributed to several factors, including the random selection and number of samples, sex, the ethnic background of the study participants, and differences in the chosen examination methods^12,25,26^. While factors like wear, caries, occlusal trauma, and the time of eruption can affect root canal anatomy, age has been shown to be a crucial factor in causing morphological changes in the root canal system’s volume, even as demonstrated by micro-CT^27^. Overall, the hypothesis of this study, i.e. the occurrence of different RCCs of MxCs in a Swiss-German population, was confirmed by the results mentioned.

One main physiological foramen was present in 79.4%, followed by two in 18.6% and three in 2.1%. In 67.0% of the investigated MxC, no accessory foramina were observed. Accessory and connecting canals were detected in 52.6% of the samples. The majority (50.5%) were in the apical root third, 2.1% in the middle, and none in the coronal third. These results match with Plascencia et al. where 43.75% of MxCs had accessory canals in the apical 5 mm of the root^19^.

The clinician needs to be aware of the presence of complex morphologies in the RCC as well as potential accessory canals that may not be mechanically accessible and prepared during the root canal preparation of MxCs. This emphasizes the value of a better understanding of root canal anatomy/configuration to ultimately make both better clinical decisions, such as chemical irrigation before obturation, to increase the success of endodontic treatments. The results emphasize the need to consider complex morphological variants as well as accessory foramina and canals when planning endodontic treatments. This could be achieved by using specific irrigation protocols and three-dimensional obturation techniques. The application of an adequate irrigation protocol and an appropriate obturation technique for each case, thus has a significant impact on the success of endodontic treatments. Regarding the description of findings from micro-CT studies and standardization of imaging techniques, the further development of classification systems should also be mentioned to describe the fine anatomical details^28,29^.

### Limitations and strengths

One limitation of this study on the Swiss-German population examined is that no information was available on the sex or age of the samples. Another limiting factor is the lack of differentiation between teeth on the right and left side of the jaw, although anatomical asymmetries cannot be ruled out. Factors such as masticatory forces or tooth abrasion, lifestyle habits and environmental influences that could affect root canal morphology in the long term were not examined either. The sample size should also be mentioned at this point. On the one hand, a sufficient sample size should be ensured by performing a sample size calculation and, in comparison with other studies on the same group of teeth in MxCs, is approximately in the range of the most common sample size of other comparable studies. However, there are numerous studies that have examined a higher number of samples but only using CBCT^12^. The only study that also used micro-CT^19^ was based on only one-third of the Swiss-German population examined in this study, although the Swiss-German population has not yet been mentioned or examined in the literature.

Although micro-CT provides the most accurate information on root canal morphology, it can only be used for ex vivo analyses for research and not for clinical use. Despite the outstanding resolution and together with the software, also three-dimensional representation of the teeth, it should be mentioned that despite completely intact samples, storage times of the teeth at very high resolution can lead to artifacts and therefore to misinterpretations. Metal fillings or other dental materials can also cause artifacts that impair the image quality. Due to the high radiation exposure, this method cannot be used in vivo; CBCT is a suitable alternative for these examinations. As a non-destructive and reproducible method, micro-CT analysis contributes to a better understanding of the complex morphology of root canal systems. This provides important insights that can lead to improved endodontic treatment.

## Conclusions

- The most frequently observed RCCs of MxCs of the Swiss-German population were 1-1-1/1 (77.3%), 1-1-1/2 (14.4%), 1-1-2/2 (4.1%), and 1-1-1/3 as well as 1-2-1/1 with 2.1% each.
- One main physiological foramen was present in 79.4%, two in 18.6%, and three in 2.1%.
- 52.6% of the samples had accessory canals, most of them in the apical region (50.5%).

These results emphasize the importance of thorough preoperative diagnostics, as complex root canal configurations and accessory canals—particularly in the apical third—can pose challenges for preparation, disinfection, and obturation. To achieve optimal treatment outcomes for MxCs with challenging RCCs, clinicians should incorporate adequate imaging techniques, effective irrigation protocols, and three-dimensional obturation methods, especially since more than half of the examined canals contained accessory canals and over one-fifth exhibited complex RCCs.

## Data Availability

Data is provided within the manuscript.
